# Molecular data reveals a new holomorphic marine fungus, *Halobyssothecium estuariae*, and the asexual morph of *Keissleriella phragmiticola*

**DOI:** 10.1080/21501203.2019.1700025

**Published:** 2019-12-09

**Authors:** Bandarupalli Devadatha, Mark S. Calabon, Pranami D. Abeywickrama, Kevin D. Hyde, E.B. Gareth Jones

**Affiliations:** a Center of Excellence in Fungal Research, Mae Fah Luang University, Chiang Rai, Thailand; b Institute of Plant and Environment Protection, Beijing Academy of Agriculture and Forestry Sciences, Beijing, China; c Department of Botany and Microbiology, College of Science, King Saud University, Riyadh, Kingdom of Saudi Arabia; d Nantgaredig, Southsea, UK

**Keywords:** 1 new taxon, salt marsh plants, marine fungi, cryptic species, taxonomy

## Abstract

This study introduces a novel holomorphic marine fungal species, *Halobyssothecium estuariae* (Lentitheciaceae, Pleosporales), from dead *Phragmites communis*. The new species has semi-immersed, subglobose or ellipsoidal, papillate, conical ascomata, clavate to subcylindrical, short pedicellate asci and 3-septate, fusoid to ellipsoidal ascospores with rounded ends, pale brown to dark brown central cells and hyaline end cells. The asexual morph has multiseptate, filiform, intercalary, catenate, branched chlamydospores that resemble *Xylomyces*. The asexual morph of *Keissleriella phragmiticola* based on combined LSU, SSU, ITS and TEF1 sequence analyses is reported. The role of molecular identification in delineating cryptic species are also discussed.

## Introduction

Salt marshes are worldwide costal marine ecosystems (Allen and Pye 1992; Simas et al. 2001), with diverse halophytic macrophytes such as *Spartina* spp., *Juncus roemerianus, Suaeda maritima, S. monoica, Phragmites* spp. and sea grass species of *Halodule, Thalassia* and *Zostera* occur (Teal 1962; Christian et al. 1990; Newell et al. 1995; Van Ryckegem et al. 2006; Calado and Barata 2012; Dayarathne et al. 2019). Salt marshes are distributed worldwide and are recognised as a most productive costal ecosystem, with a vital role in nutrient recycling and shoreline protection (Gessner and Kohlmeyer 1976; Newell et al. 1995; Newell 1996; Calado et al. 2015, 2019). Studies on salt marsh plants, such as *Spartina alterniflora, Juncus roemerianus* and *Phragmites australis*, have been shown to support a great diversity of marine fungi akin to many mangrove plants (Fell and Hunter 1979; Cuomo et al. 1982, 1985; Poon and Hyde 1998; Barata 2002; Kohlmeyer and Volkmann-Kohlmeyer 2002; Wong and Hyde 2002; Van Ryckegem and Verbeken 2005; Calado and Barata 2012; Jones et al. 2019).

The genus *Phragmites* includes predominant perennial grasses found in marine coastal environments worldwide, throughout temperate and tropical regions and *Phragmites australis* has been widely studied for marine fungi (Poon and Hyde 1998; Wong and Hyde 2002; Van Ryckegem and Verbeken 2005). More than 300 fungi have been reported in association with this plant (Wong and Hyde 2001, 2002; Calado et al. 2015; Goonasekara et al. 2019), of which 109 species were recorded from intertidal marshes in Hong Kong. Poon and Hyde (1998) described three new species *Massarina phragmiticola, Phomatospora phragmiticola* and *Cytoplacosphaeria phragmiticola* from this plant and Wong et al. (1998) introduced the novel genus *Phragmitensis* typified by *P. marina*. Karunarathna et al. (2017) introduced a new aquatic genus and species *Yunnanensis phragmitis* collected on *Phragmites australis* from Dali Lake, Yunnan Province in China, while Wanasinghe et al. (2018) described *Keissleriella phragmiticola* on *Ph. communis* collected from Poole, Dorset, U.K.

The family Lentitheciaceae, typified by *Lentithecium fluviatile* was established by Zhang et al. (2012) to introduce selected massarina-like species (Zhang et al. 2009, 2012; Hyde et al. 2013, 2016; Tibpromma et al. 2017). Members of this family are saprobic on herbaceous and woody plants in various habitats. They have globose to lenticular ascomata with a short-papilla, asci with a short pedicel and ascospores that are fusiform to cylindrical, filiform in some species, 1–3-septate or muriform in a few species, surrounded by a mucilaginous sheath or extended appendage-like sheath and asexual morphs producing stagonospora-like or dendrophoma-like sporulating structures (Wanasinghe et al. 2014; Luo et al. 2016; Su et al. 2016). Currently, 12 genera have been circumscribed within this family: *Darksidea* (Knapp et al. 2015), *Halobyssothecium* (Dayarathne et al. 2018), *Katumotoa, Keissleriella, Lentithecium, Murilentithecium* (Wanasinghe et al. 2014), *Neoophiosphaerella, Phragmocamarosporium, Poaceascoma, Setoseptoria*, and *Tingoldiago* (Tanaka et al. 2015) and *Towyspora* (Li et al. 2016).

The ascomycete *Halobyssothecium obiones* has a chequered history assigned to various genera and families and is reported from a variety of host plants. Assigned initially as two separate species: *Pleospora obiones* by Crouan and Crouan (1867) and *Leptosphaeria discors* by Saccardo and Ellis (1882). Subsequently, they have been assigned to various genera: *Metasphaeria* (Saccardo 1883), *Heptameria* (Cooke 1889), *Passeriniella* (Apinis and Chesters 1964; Hyde and Mouzouras 1988; Khashnobish and Shearer 1996a) and more recently, based on a multi locus phylogenetic study to *Halobyssothecium* (Dayarathne et al. 2018). *Didymosphaeria spartinae* is also included in synonymy with *H. obiones* (Grove 1933). As a consequence, it has also been referred to different families in the Dothideomycetes. Studies of *Halobyssothecium obiones/Leptosphaeria discors* reveal significant differences in the ascospore dimensions: in most collections, they are 24–38 × 8–14 μm (Dayarathne et al. 2018), while others measure 38–56 × 16–22 μm (Jones 1962; Cavaliere 1968; Webber 1970). Kohlmeyer and Kohlmeyer (1979) provided a description of *Leptosphaeria discors* based on examination of the type material in Herb Crouan at Concarneau, France and the account of spermagonia for this species by Wagner (1965). Kohlmeyer and Kohlmeyer (1979) concluded that because of these differences in ascospore measurements “it appears to be a new species in need of thorough examination”. The results of our study and the above observations suggest that *Halobyssothecium obiones* is a species complex.

*Halobyssothecium obiones* has a worldwide distribution in temperate regions and occurs as a saprobe of *Agropyron junceiforme, Halimione portulacoides, Spartina* spp., on intertidal wood, bamboo, and exposed test panels of *Betula pubescens* and *Fagus sylvatica* (Kohlmeyer and Kohlmeyer 1979). Dayarathne et al. (2018) recollected *Byssothecium* (*= Halobyssothecium*) *obiones* from *Spartina* culms which enabled phylogenetic studies. Based on a multi-locus phylogenetic analyses and morphological observations, Dayarathne et al. (2018) showed that their collection grouped in Lentitheciaceae and proposed a novel genus *Halobyssothecium* typified by *H. obiones* (= *Byssothecium obiones*).

The present study aims to examine if *Halobyssothecium obiones* is a species complex and introduces a new species *Halobyssothecium estuariae* with both sexual and asexual morphs that are found on dead culms of *Ph. communis*. The genus *Keissleriella*, typified by *Keissleriella aesculis*, was introduced by Höhnel (1919). It is characterised by ascomata with ostiolar necks filled with black setae and one to multi-septate, hyaline ascospores with a pycnidial coelomycetous asexual morph producing 0–3 septate hyaline conidia (Barr 1990; Tanka et al. 2015). In our ongoing studies, we have found a coelomycetous asexual morph inhabiting *Ph. communis* and identified as *Keissleriella phragmiticola* (Wanasinghe et al. 2018) based on sequence data. The asexual morph is illustrated and supported by molecular evidence.

## Materials and methods

### Sample collection, isolation and morphological studies

Dead and decaying culms of *Phragmites communis* and *Spartina* sp. were collected from Slebech Estuary, Pembrokeshire, UK. and the Ketch Nature Reserve, Hayling Island, UK. Specimens were placed in a Ziplock plastic bags and incubated at room temperature in the laboratory. Specimens were examined under a Leica EZ4 stereo zoom microscope. Hand sections of the ascomata were made and the centrum contents were taken out with the aid of a needle and fixed in sterile distilled water. The microscopic characters were photographed using Carl Zeiss Discovery V8 stereo-microscope fitted with Axiocam and Nikon ECLIPSE TiU upright microscope with DIC objectives connected to Nikon DS-Fi2 digital camera. The morphological measurements were taken by means of Tarosoft (R) Image Frame Work program v. 0.9.7. The pictures in the photo plates were arranged by using Adobe Photoshop CS6 Extended v. 13.0

Isolates were obtained by using a single spore isolation method as described in Choi et al. (1999) using sea salt agar media. The germinating ascospores from ascomycetes and conidia from asexual morphs were transferred to sea salt malt extract agar media (SMEA) plates and incubated at 25°C for 10 to 20 days with regular observations. The herbaria and the axenic type cultures were deposited in Mae Fah Luang University herbarium and Mae Fah Luang University Culture Collection (MFLUCC), Chiang Rai, Thailand. Facesoffungi and Index Fungorum numbers were acquired as elucidated in Jayasiri et al. (2015) and Index Fungorum (2019).

### DNA extraction, PCR amplification and sequencing

The hyphal mass from freshly grown colonies were scraped by using a sterile lancet and transferred to a 1.5 ml Eppendorf tube (Christian et al.  1990). Total genomic DNA was isolated by following CTAB methods described by Jeewon et al. (2002) and Suwannarach et al. (2010). Four loci were amplified by employing well-known primer pairs: ITS4 and ITS5 to amplify ITS region and nuclear small subunit rDNA region with NS1 and NS4 (White et al. 1990). Nuclear large subunit rDNA (LSU) was amplified using LR0R and LR5 (Vilgalys and Hester 1990). The translation elongation factor 1-alpha gene (TEF-1α) was amplified using primers EF1–983F and EF1–2218R (Rehner and Buckley 2005).

PCR reactions were carried out using volume of 50 µL composed of 5 µL of *Ex Taq* buffer, 4 µL of dNTP mixture, 1 µL of each primer, 1 µL (50–100 ng) genomic DNA, 0.3 µL of *TaKaRa Ex Taq* ™ polymerase and the remaining volume with that of double distilled water. PCR amplification conditions were set as follows; an initial denaturation at 95°C for 3 min, followed by 35 cycles of denaturation at 95°C for 30 s, primer annealing at 54°C for SSU; 52°C for ITS, LSU and TEF1α, primer extension at 72°C for 1 min, and a final extension step at 72°C for 10 min. All PCR products were visualised on a 1.2% agarose gel stained with ethidium bromide and purified by Qiagen purification kit (Qiagen, USA) following the manufacturer’s procedure. PCR products of different genes were then sequenced with primers stated above by Biomed company, Beijing, China.

### Sequence alignment and phylogenetic analyses

Sequences of different gene regions from both forward and reverse primers were assembled to obtain a consensus sequence with BioEdit v.7.0.5.2 (Hall 1999). Based on the mega BLAST searched in NCBI two strains were assigned to *Halobyssothecium* and one strain to *Keissleriella*, Lentitheciaceae. The taxa for the phylogenetic analyses were downloaded from GenBank and those identified from a recent study of *Halobyssothecium* (Dayarathne et al. 2018). Multiple sequence alignments for different gene regions were generated online at MAFFT server (http://mafft.cbrc.jp/alignment/server/) (Katoh and Standley 2013) and alignments were manually adjusted using BioEdit, wherever essential. The individual sequence datasets (LSU, SSU, ITS, TEF-1α) were concatenated using BioEdit v.7.0.5.2 (Hall 1999).

For Maximum Likelihood and Bayesian analyses, MrModeltest v. 2.3 (Nylander 2004) was used to determine the best-fit model of nucleotide evolution for the dataset. GTR+I + G model was selected.

**Figure 1.**
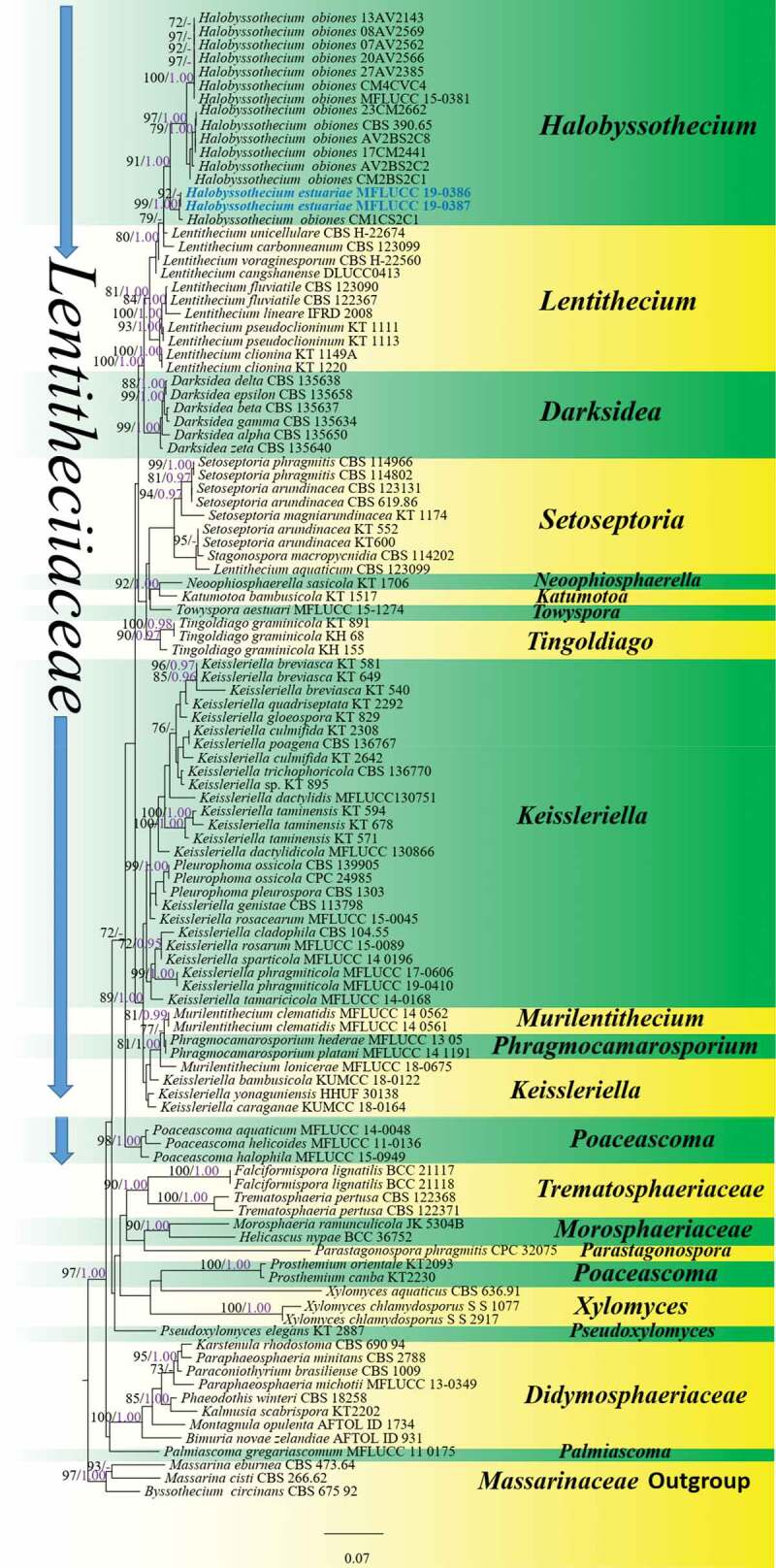
Phylogram based on analysis of the combined dataset of LSU, SSU, ITS and TEF-1α sequence data. Bootstrap support values for ML (>70%) and BYPP values (>0.95) are given above each branch. The new isolates are represented in blue. The tree is rooted to *Byssothecium circinans* CBS 67592, *Massarina cisti* CBS 26,662 and *Massarina eburnea* CBS 473.64 (Massarinaceae). Bar = 0.07 estimated number of nucleotide substitutions per site per branch.

RAxML-HPC2 on XSEDE (8.2.8) (Stamatakis et al. 2008; Stamatakis 2014) in the CIPRES Science Gateway platform (Miller et al. 2010) was used to build a maximum likelihood (ML) tree using GTR+I + G model of evolution. Maximum Likelihood bootstrap values greater than 70% were given above each node for (Figure 1).

Bayesian analysis was implemented by using MCMC sampling in MrBayes v. 3.1.2 (Huelsenbeck and Ronquist 2001) to evaluate Bayesian posterior probabilities by Markov Chain Monte Carlo sampling (MCMC) with two runs and four chains (Rannala and Yang 1996; Zhaxybayeva and Gogarten 2002). A total of 10,000,000 MCMC generations and trees were sampled every 1000th generation, resulting in a total of 10,000 trees. First 1000 trees were discarded as burn-in phase and the remaining 9000 trees were used to calculate posterior probabilities in the majority rule consensus tree. BYPP greater than 0.95 are given above each node (Figure 1).

The phylogenetic trees were viewed in FigTree v1.4.0 program (Rambaut 2012) and restructured in Microsoft Power point (2016) and Adobe Illustrator® CS5 (Version 15.0.0, Adobe®, San Jose, CA). New sequences generated in the present study were deposited in GenBank (Table 1) and the sequence alignments were deposited at TreeBASE (www. treebase.org) under the accession number: 25,320

**Table 1.** Genbank accession numbers for taxa used in the phylogenetic analyses.

		DDBJ/GenBank/EMBL accession no.^a^				
Taxon name	Strain no.	LSU	SSU	ITS	TEF-1α	References
*Bimuria novaezelandiae*	AFTOL-ID 931	–	–	–	DQ471087	Spatafora et al. (2006)
*Byssothecium circinans*	CBS67,592	GU205217	GU205235	–	GU349061	Hu et al. (2009)
*Darksidea alpha*	CBS 135,650	KP184019	KP184049	NR137619	KP184166	Vu et al. (2018)
*Darksidea beta*	CBS 135,637	KP184023	KP184074	NR137957	KP184189	Vu et al. (2018)
*Darksidea delta*	CBS 135,638	–	–	NR137075	–	Vu et al. (2018)
*Darksidea epsilon*	CBS 135,658	KP184029	KP184070	NR137959	KP184186	Vu et al. (2018)
*Darksidea gamma*	CBS 135,634	KP184031	KP184073	NR137587	KP184188	Vu et al. (2018)
*Darksidea zeta*	CBS 135,640	KP184013	KP184071	NR137958	KP184191	Vu et al. (2018)
*Falciformispora lignatilis*	BCC 21,117	GU371835	GU371835	KF432942	GU371820	Schoch et al. (2009)
*Falciformispora lignatilis*	BCC 21,118	GU371826	GU371835	GU371835	GU371820	Schoch et al. (2009)
***Halobyssothecium estuariae***	**MFLUCC 19-0386**	**MN598871**	**MN598868**	**MN598890**	**MN597050**	**This study**
***Halobyssothecium estuariae***	**MFLUCC 19-0387**	**MN598872**	**MN598869**	**MN598891**	**MN597051**	**This study**
*Halobyssothecium obiones*	07AV2562	–	–	KX263858	–	Calado et al. (2015)
*Halobyssothecium obiones*	08AV2569	–	–	KX263859	–	Calado et al. (2015)
*Halobyssothecium obiones*	13AV2143	–	–	KX263860	–	Calado et al. (2015)
*Halobyssothecium obiones*	17CM2441	–	–	KX263861	–	Calado et al. (2015)
*Halobyssothecium obiones*	20AV2566	–	–	KX263862	–	Calado et al. (2015)
*Halobyssothecium obiones*	23CM2662	–	–	KX263863	–	Calado et al. (2015)
*Halobyssothecium obiones*	27AV2385	–	–	KX263864	–	Calado et al. (2015)
*Halobyssothecium obiones*	AV2BS2C2	–	–	KX263805	–	Calado et al. (2015)
*Halobyssothecium obiones*	AV2BS2C8	–	–	KX263806	–	Calado et al. (2015)
*Halobyssothecium obiones*	CBS 390.65	MH870267	–	MH858628	–	Khashnobish and Shearer (1996b)
*Halobyssothecium obiones*	CM1CS2C1	–	–	KX263807	–	Calado et al. (2015)
*Halobyssothecium obiones*	CM2BS2C1	–	–	KX263808	–	Calado et al. (2015)
*Halobyssothecium obiones*	CM4CVC4	–	–	KX263809	–	Calado et al. (2015)
*Halobyssothecium obiones*	MFLUCC 15-0381	MH376744	MH376745	MH377060	MH376746	Dayarathne et al. (2018)
*Helicascus nypae*	BCC36752	GU479789	GU479755	–	GU479855	Suetrong et al. (2009)
*Kalmusia scabrispora*	KT2202	AB524594	AB524453	–	AB539107	Suetrong et al. (2009)
*Karstenula rhodostoma*	CBS69094	GU301821	GU296154	–	GU349067	Schoch et al. (2009)
*Katumotoa bambusicola*	KT1517a	AB524595	AB524454	LC014560	AB539108	Tanaka et al. (2009)
*Keissleriella bambusicola*	KUMCC18-0122	MK995880	MK995878	MK995881	–	Jiang et al. (2019)
*Keissleriella breviasca*	KT540	AB807586	AB797296	–	AB808565	Tanaka et al. (2015)
*Keissleriella breviasca*	KT581	AB807587	AB797297	–	AB808566	Tanaka et al. (2015)
*Keissleriella breviasca*	KT649	AB807588	AB797298	–	AB808567	Tanaka et al. (2015)
*Keissleriella caraganae*	KUMCC18-0164	MK359439	MK359444	NR164447	MK359073	Phookamsak et al. (2019)
*Keissleriella cladophila*	CBS104.55	GU301822	GU296155	–	GU349043	Schoch et al. (2009)
*Keissleriella culmifida*	KT2308	AB807591	AB797301	LC014561	–	Tanaka et al. (2015)
*Keissleriella culmifida*	KT2642	AB807592	AB797302	LC014562	–	Tanaka et al. (2015)
*Keissleriella dactylidicola*	MFLUCC13-0866	KT315506	KT315505	–	KT315507	Ariyawansa et al. (2015)
*Keissleriella dactylidis*	MFLUCC 13-0751	KP197668	KP197666	KP197667	KP197669	Singtripop et al. (2015)
*Keissleriella genistae*	CBS 113,798	GU205222	GU205242	–	–	Hu et al. (2009)
*Keissleriella gloeospora*	KT829	AB807589	AB797299	LC014563	–	Vu et al. (2018)
*Keissleriella phragmiticola*	MFLUCC 17-0779	MG829014	–	MG828904	–	Wanasinghe et al. (2018)
***Keissleriella phragmiticola***	**MFLUCC 19-0410**	**MN598873**	**MN598870**	**MN598892**	**MN607978**	**This study**
*Keissleriella poagena*	CBS136767	KJ869170	–	KJ869112	–	Crous et al. 2014
*Keissleriella quadriseptata*	KT2292	AB807593	AB797303	AB811456	AB808572	Tanaka et al. (2015)
*Keissleriella rosacearum*	MFLUCC 15-0045	MG829015	MG829123	NG_063684	–	Wanasinghe et al. (2018)
*Keissleriella rosarum*	MFLUCC 15-0089	MG828905	NG063685	–	–	Wanasinghe et al. (2018)
*Keissleriella* sp.	KT895	AB807590	AB797300	–	AB808569	Vu et al. (2018)
*Keissleriella sparticola*	MFLUCC 14-0196	KP639571	–	–	–	Liu et al. 2015
*Keissleriella tamariciola*	MFLUCC 14-0168	KU900300	–	KU900328	–	Thambugala et al. 2017
*Keissleriella taminensis*	KT571	AB807595	AB797305	LC014564	AB808574	Tanaka et al. (2015)
*Keissleriella taminensis*	KT594	AB807596	AB797306	–	–	Tanaka et al. (2015)
*Keissleriella taminensis*	KT678	AB807597	AB797307	LC014565	AB808575	Tanaka et al. (2015)
*Keissleriella trichophoricola*	CBS 136,770	KJ869171	–	KJ869113	–	Vu et al. (2018)
*Keissleriella yonaguniensis*	HHUF 30,138	NG059402	NG064856	AB811457	–	Tanaka et al. (2015)
*Lentithecium aquaticum*	CBS 123,099	GU301823	GU296156	–	GU349068	Suetrong et al. 2009
*Lentithecium cangshanense*	DLUCC 0143	KU991149	–	KU991150	–	Su et al. (2016)
*Lentithecium carbonneanum*	CBS 144,076	MH069699	–	NR158534	–	Crous et al. (2018)
*Lentithecium clionina*	KT1149A	AB807540	AB797250	LC014566	AB808515	Tanaka et al. (2015)
*Lentithecium clionina*	KT1220	AB807541	AB797251	–	AB808516	Tanaka et al. (2015)
*Lentithecium fluviatile*	CBS 122,367	FJ795451	FJ795493	–	GU456290	Schoch et al. (2009)
*Lentithecium fluviatile*	CBS 123,090	FJ795450	FJ795492	–	–	Zhang et al. (2009)
*Lentithecium lineare*	IFRD 2008	FJ795435	FJ795478	–	–	Zhang et al. (2009)
*Lentithecium pseudoclioninum*	KT1111	AB807544	AB797254	AB809632	AB808520	Tanaka et al. (2015)
*Lentithecium pseudoclioninum*	KT1113	AB797255	AB807545	AB809633	AB808521	Tanaka et al. (2015)
*Lentithecium voraginesporum*	CBS H-22,560	NG066171	–	NG063065	–	Hyde et al. (2016)
*Lentithecium unicellulare*	CBS H-22674	NG058261	–	KX505374.	–	Hyde et al. (2016)
*Massarina cisti*	CBS 266.62	FJ795447	FJ795490	LC014568	AB808514	Zhang et al. 2009
*Massarina eburnea*	CBS 473.64	GU301840	GU296170	–	GU349040	Schoch et al. (2009)
*Montagnula opulenta*	AFTOLID 1734	DQ678086	AF164370	–	–	Schoch et al. (2006)
*Morosphaeria ramunculicola*	JK 5304B	GU479794	GU479760	–	–	Suetrong et al. 2009
*Murilentithecium clematidis*	MFLUCC 14-0561	KM408758	KM408759	KM408756	KM454444	Wanasinghe et al. (2014)
*Murilentithecium clematidis*	MFLUCC 14-0562	KM408760	KM408761	KM408757	KM454445	Wanasinghe et al. (2014)
*Murilentithecium lonicerae*	KUMCC 18-0168	MK359441	MK359446	MK359436	MK359075	Phookamsak et al. (2015)
*Neoophiosphaerella sasicola*	KT1706	AB524599	AB524458	LC014577	AB539111	Tanaka et al. (2009)
*Palmiascoma gregariascomum*	MFLUCC 11-0175	KP744495	KP753958	KP744452	–	Liu et al. (2015)
*Paraconiothyrium brasiliense*	CBS 100,299	JX496124	AY642523	JX496011	AY642531	Verkley et al. (2014)
*Paraphaeosphaeria michotii*	MFLUCC 13-0349	KJ939282	KJ939285	KJ939279	–	Ariyawansa et al. (2014)
*Paraphaeosphaeria minitans*	CBS 122,788	EU754173	EU754074	–	GU349083	De Gruyter et al. (2009)
*Parastagonospora phragmitis*	CPC 32,075	MK540029	–	NR164454	MK540152	Marin-Felix et al. (2019)
*Phaeodothis winteri*	CBS 18,258	GU301857	GU296183	–	–	Schoch et al. (2009)
*Phragmocamarosporium hederae*	MFLUCC 13-0552	KP842915	KP842918	–	–	Wijayawardene et al. (2015)
*Phragmocamarosporium platani*	MFLUCC 14-1191	KP842915	KP842918	–	–	Wijayawardene et al. (2015)
*Pleurophoma ossicola*	CBS 139,905	KR476769	–	KR476736	–	Crous et al. (2015)
*Pleurophoma ossicola*	CPC 24,985	KR476770	–	NR137992	–	Crous et al. (2015)
*Pleurophoma pleurospora*	CBS130329	JF740327	–	–	–	De Gruyter et al. (2013)
*Poaceascoma aquaticum*	MFLUCC 14-0048	KT324690	KT324691	–	–	Luo et al. (2016)
*Poaceascoma halophila*	MFLUCC 15-0949	MF615399	MF615400	–	–	Hyde et al. (2017)
*Poaceascoma helicoides*	MFLUCC 11-0136	KP998462	KP998463	KP998459	KP998461	Phookamsak et al. (2015)
*Prosthemium canba*	KT2230	AB553766	–	AB554096	–	Tanaka et al. (2010)
*Prosthemium orientale*	KT2093	AB553750	AB553642	AB554081	–	Tanaka et al. (2010)
*Pseudoxylomyces elegans*	KT2887	AB807598	AB797308	LC014593	–	Tanaka et al. (2015)
*Setoseptoria arundinacea*	CBS 123,131	GU456320	GU456298	–	GU456281	Zhang et al. (2009)
*Setoseptoria arundinacea*	CBS 619.86	GU301824	GU296157	–	–	Schoch et al. (2009)
*Setoseptoria arundinacea*	KT552	AB807574	AB797284	–	AB808550	Tanaka et al. (2015)
*Setoseptoria arundinacea*	KT600	AB807575	AB797285	LC014595	AB808551	Tanaka et al. (2015)
*Setoseptoria magniarundinacea*	KT1174	AB807576	AB797286	LC014596	AB808552	Tanaka et al. (2015)
*Setoseptoria phragmitis*	CBS 114,802	KF251752	–	KF251249	KF253199	Quaedvlieg et al. (2013)
*Setoseptoria phragmitis*	CBS 114,966	KF251753	–	KF251250	KF253200	Quaedvlieg et al. 2013
*Stagonospora macropycnidia*	CBS 114,202	GU301873	GU296198	–	GU349026	Schoch et al. (2009)
*Tingoldiago graminicola*	KH155	AB521745	AB521728	LC014599	AB808562	Hirayama et al. (2010)
*Tingoldiago graminicola*	KH68	AB521743	AB521726	LC014598	AB808561	Hirayama et al. (2010)
*Tingoldiago graminicola*	KT891	AB521744	AB521727	–	AB808563	Hirayama et al. (2010)
*Towyspora aestuari*	MFLUCC 15-1274	KU248852	KU248853	NR148095	–	Li et al. (2016)
*Trematosphaeria pertusa*	CBS 122,368	FJ201990	FJ201991	NR132040	KF015701	Zhang et al. (2008)
*Trematosphaeria pertusa*	CBS 122,371	GU301876	GU348999	KF015669	KF015702	Schoch et al. (2009)
*Xylomyces aquaticus*	CBS 636.91	–	–	FJ887921	–	Prihatini et al. (2008)
*Xylomyces chlamydosporus*	S S 1077	–	–	FJ887918	–	Suetrong et al. (2011)
*Xylomyces chlamydosporus*	S S 2917	–	JN819291	FJ887919	–	Suetrong et al. (2011)

^a^ Sequences generated in the present study are indicated in bold.

BCC: BIOTEC Culture Collection, Bangkok, Thailand; BJFUCC: Beijing Forestry University Culture Collection; CBS: Centraalbureau Voor Schimmelcultures, Utrecht, The Netherlands; Dali University Culture Collection (DLUCC); JF: Jacques Fournier; JK: J. Kohlmeyer; KH: K. Hirayama, KT: Kazuaki Tanaka, MAFF: Ministry of Agriculture, Forestry and Fisheries, Japan; MFLUCC: Mae Fah Luang University Culture Collection, Chiangrai, Thailand; NFCCI: National Fungal Culture collection of India; SS: Satinee Suetrong Figure 1. Phylogram based on analysis of the combined dataset of LSU, SSU, ITS and TEF-1α sequence data. Bootstrap support values for ML (>70%) and BYPP values (>0.95) are given above each branch. The new isolates are represented in blue. The tree is rooted to *Byssothecium circinans* CBS 67,592, *Massarina cisti* CBS 26,662 and *Massarina eburnea* CBS 473.64 (Massarinaceae). Bar = 0.07 estimated number of nucleotide substitutions per site per branch.

## Results

### Phylogenetic analyses

**Figure 2.**
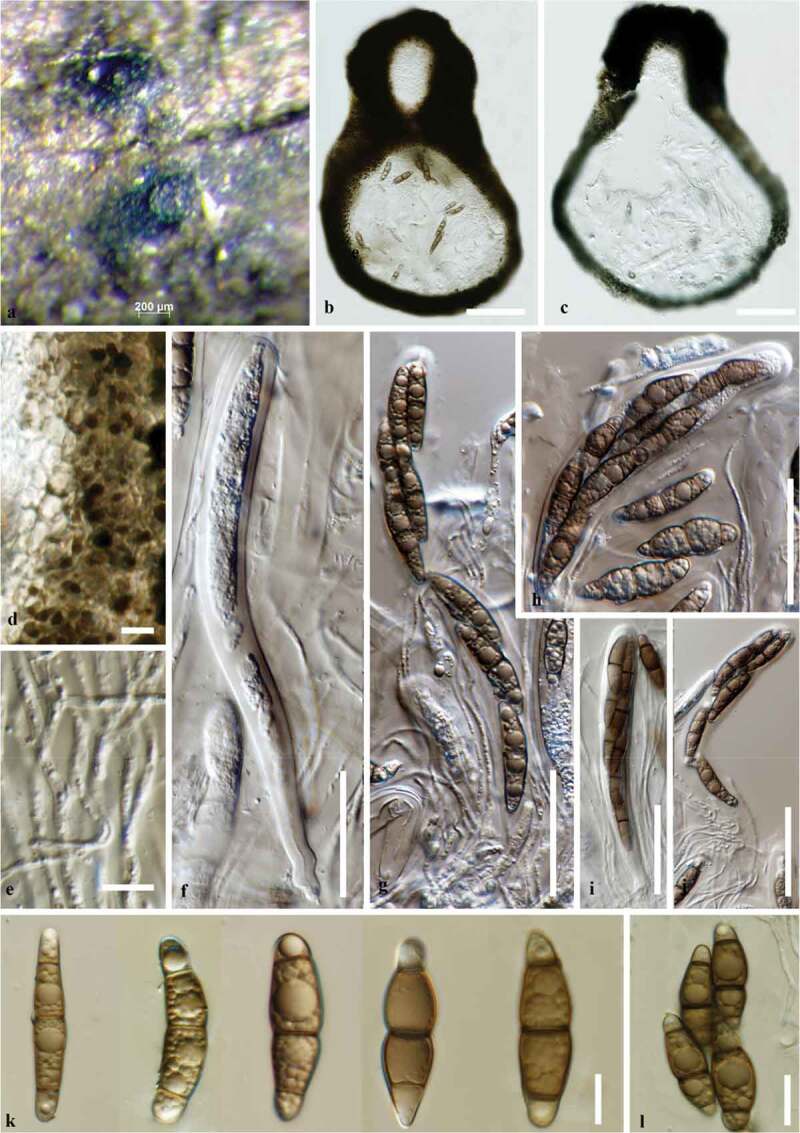
*Halobyssothecium estuariae* (MFLUCC 19–0386, holotype) (a) Appearance of ascomata on the host surface. (b, c) Vertical sections through ascomata (d) Peridium (e) Pseudoparaphyses (f–j) Immature and mature asci (k,l) Ascospores with central brown and hyaline end cells. Scale bars: b–c = 100 µm, f–j = 50 µm, d, e, k–l = 10 µm.

The combined LSU, SSU, ITS and TEF-1α gene dataset comprised 110 taxa with 3397 nucleotide characters from taxa belonging to the families Aliquandostipitaceae, Bambusicolaceae, Lentitheciaceae, Massarinaceae, Morosphaeriaceae, Didymosphaeriaceae, Pleomassariaceae and Trematosphaeriaceae were performed. *Byssothecium circinans* CBS 67,592, *Massarina cisti* CBS 26,662 and *Massarina eburnea* CBS 473.64 were selected as outgroups (Table 1). RAxML analysis of the combined dataset yielded the best tree (Figure 2) with a final ML optimisation likelihood value of −23,667.157059. The matrix had 1338 different alignment patterns, with 31.17% of undetermined characters or gaps. Base frequencies were: A = 0.239291, C = 0.247966, G = 0.271866, T = 0.240877. The analysis assessed a proportion of transitions and transversions in which AC = 1.162556, AG = 2.249760, AT = 1.422977, CG = 1.061904, CT = 6.568313, GT = 1.000000; Invariable sites composed 0.504689 of the datasets and the gamma distribution shape parameter was 0.476850. Trees resulting from ML and BIPP were similar in topology.

The combined multi-locus phylogenetic analyses showed that isolates of the new taxon *Halobyssothecium estuariae* clustered together and shared a sister relationship with a clone of *Halobyssothecium obiones* (CM1CS2C1) in a monophyletic clade with significant bootstrap support (99% ML and 1.00 BYPP). The recently epitypified *Halobyssothecium obiones* MFLUCC 15–0381, and other strains, together formed a monophyletic clade with strong bootstrap support (100% ML and 1.00 BYPP) and share a sister group relationship with *Halobyssothecium estuariae.*

***Taxonomy***

**Figure 3.**
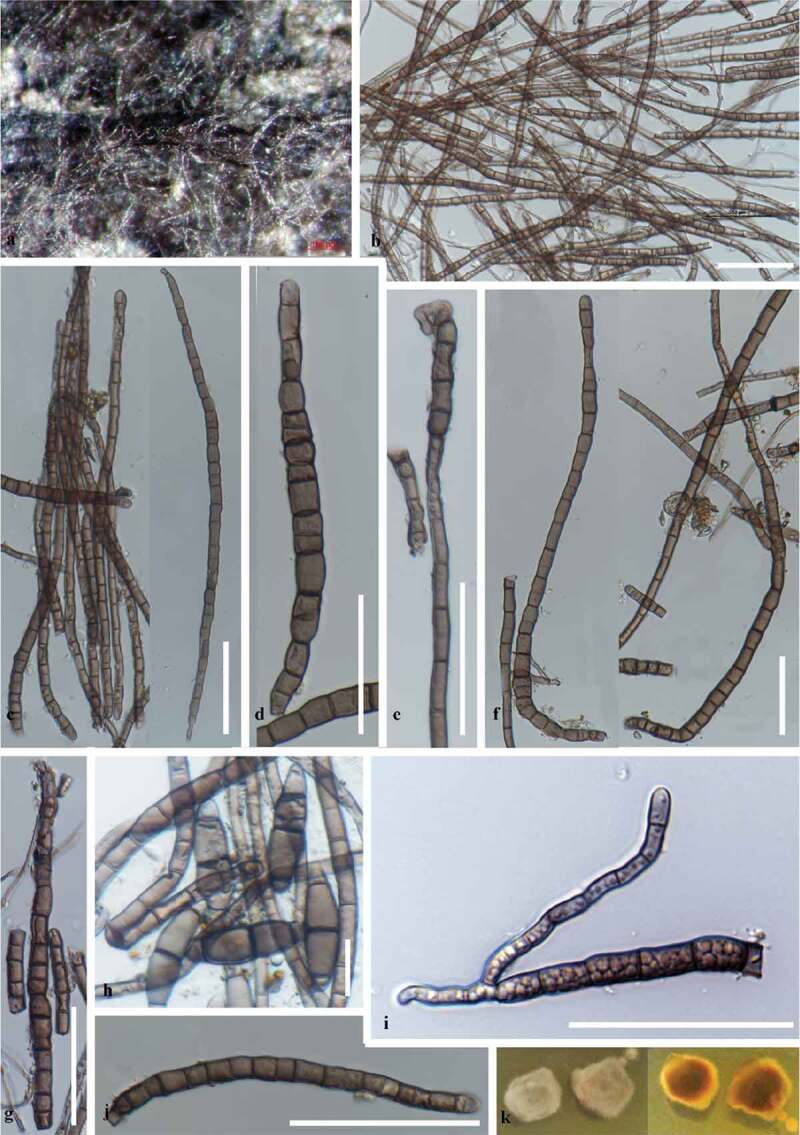
*Halobyssothecium estuariae* (MFLUCC 19–0387, asexual morph holotype) (a) Colonies on the host surface. (b-g, j, i) Chlamydospores (h) Liberated ascospores on host along with chlamydospores. (k) Cultures on SMEA (upper, lower). Scale bars: b = 100 µm, c–g, j, i = 50 µm, h = 10 µm.

***Halobyssothecium estuariae*** B. Devadatha, Calabon, K.D. Hyde and E.B.G Jones, **sp. nov**. Figures 2 and 3*Index Fungorum number*: IF556892, *Facesoffungi number*: FOF 06769

#### Etymology

In reference to the estuarine habitat where the fungus was found.

#### Description from holotype

*Saprobic* on *Phragmites communis* in estuarine habitats. **Sexual morph**: *Ascomata* 260–420 μm high, 260–350 μm diam. ($\bar{x}$= 326 × 295 μm, n = 10), immersed to semi-immersed, sub-globose or ellipsoidal, dark brown to black, carbonaceous, scattered, ostiolate, with periphyses. *Papilla* conical, 65–85 μm high, 55–85 μm wide at the top, composed of several layers of pseudoparenchymatous cells. *Peridium* 20–55 μm thick ($\bar{x}$= 42 μm, n = 10), composed of 3–4 cell layers of *textura angularis*, outermost layer of brown pseudo parenchyma and thick-walled brown cells; inner layer of elongated, hyaline cells. *Pseudoparaphyses* 1.4–2 μm wide, septate, hyaline, filiform, branched and anastomosing above the asci. *Asci* 120–235 × 10–25 μm (x̄ = 190.3 × 16 μm, n = 20), 8-spored, fissitunicate, clavate to sub cylindrical, short pedicellate with an ocular chamber. *Ascospores* 20–44 × 4–9 μm (x̄ = 32.6 ± 5.1 × 7.2 ± 1.6 μm n = 20), overlapping, uniseriate to biseriately arranged, fusoid to ellipsoidal with rounded ends, central cells that are pale brown to dark brown, hyaline end cells, 3-septate and constricted at the septa, guttulate, straight to slightly curved, lacking gelatinous sheath or appendages. **Asexual morph**: Hyphomycetous. Colonies on the natural substrate pale brown to dark brown spreading. Vegetative hyphae superficial, septate, smooth-walled, branched, brown to dark brown, 3–5 µm diam. *Conidiophores* and conidia are absent. *Chlamydospores* apical, rarely intercalary, single or in chains, branching, filamentous, filiform to narrowly fusiform straight or curved, intercalary and terminal, catenate, rarely solitary, branched, with thickened septa, smooth, 5–35 transverse septate, brown to dark brown at the septa, 35–185 × 4–8 µm.

#### Culture characteristics

Both sexual and asexual morphs have identical morphological characters in culture. Colonies on MEA, slow growing, reaching 10–25 mm diam., after 10 days of incubation at room temperature, colonies velvety, lobate edges, irregular, surface umbonate. Colony form top pale brown to reddish brown, below reddish brown with white margin.

#### Material examined

UK, Pembrokeshire, Slebech Estuary, on dead culm of *Phragmites communis* (Poaceae), 18 April 2019. E.B.G Jones. GJ619 (MFLU 19–0999, holotype), ex-type living cultures MFLUCC 19–0386 (Sexual morph), MFLUCC 19–0387 (Asexual morph).

#### Notes

*Halobyssothecium* is a monotypic genus containing the species *H. obiones*. Based on morphological characters we have assigned the sexual morph of *Halobyssothecium estuariae* (MFLUCC 19–0386) with its xylomyces-like asexual morph (MFLUCC 19–0387). The phylogenetic analysis confirmed that both the sexual morph (MFLUCC 19–0386) and asexual morph (MFLUCC 19–0387) of *Halobyssothecium estuariae* belong in Lentitheciaceae.

*Halobyssothecium estuariae* resembles the generic type *H. obiones* in possessing subglobose or ellipsoidal, carbonaceous ascomata, conical papilla and ascospores with brown central cells and hyaline end cells (Dayarathne et al. 2018). However, *Halobyssothecium estuariae* is distinct from *H. obiones* in having longer and narrow papilla (65–85 × 55–85 vs. 25–35 × 130–145 μm) and ascospores with smaller dimensions (20–44 × 4–9 vs. 28–47 × 10–18 μm). Further, the asexual morph of *Halobyssothecium estuariae* is hyphomycetous characterised by xylomyces-like chlamydospores, whereas in *H. obiones* the asexual morph produces phoma-like conidia (Kohlmeyer and Kohlmeyer 1979; Calado et al. 2015).

The asexual morph of *Halobyssothecium estuariae* is comparable to *Xylomyces* species. *Halobyssothecium estuariae* chlamydospores (MFLUCC 19–0387) shares similarities with *Xylomyces rhizophorae* in having chlamydospores that are apical, filamentous, straight or curved and dark brown. Nevertheless, *Halobyssothecium estuariae* (MFLUCC 19–0387) differs from *Xylomyces rhizophorae* in having smaller chlamydospores with 5–35 transverse septa. *Xylomyces rhizophorae* has longer chlamydospores that are 11–43 transversely septate and rarely with longitudinal or oblique septa (Kohlmeyer and Volkmann-Kohlmeyer 1998). Further, phylogenetic analyses also showed that *Halobyssothecium estuariae* (MFLUCC 19–0387) is distantly related to *Xylomyces* species. *Xylomyces rhizophorae* lacks molecular data to establish an evolutionary relationship with our taxon.

Phylogenetically, *H. obiones* and *H. estuariae* differ by 5.1% nucleotide base pair differences in ITS and 2.9% in TEF gene regions and justifies the introduction of a new species (Jeewon and Hyde 2016). Comparison of ITS nucleotide base pair differences between *Halobyssothecium obiones* (CM1CS2C1 clone) and *H. estuariae* resulted in 1.3% differences. *Halobyssothecium obiones* strain (CMICS2C1) grouping with our new species may be a misidentified *H. estuariae*. The assessment of different gene regions of both sexual morph (MFLUCC 19–0386) and asexual morph (MFLUCC 19–0387) of *Halobyssothecium estuariae* revealed 100% sequence similarity which signifies that both are identical species.

***Halobyssothecium obiones*** (P. Crouan & H. Crouan) Dayarathne, E.B.G. Jones & K.D. Hyde, Mycol. Progr. [17] (2018) Figure 4

**Figure 4.**
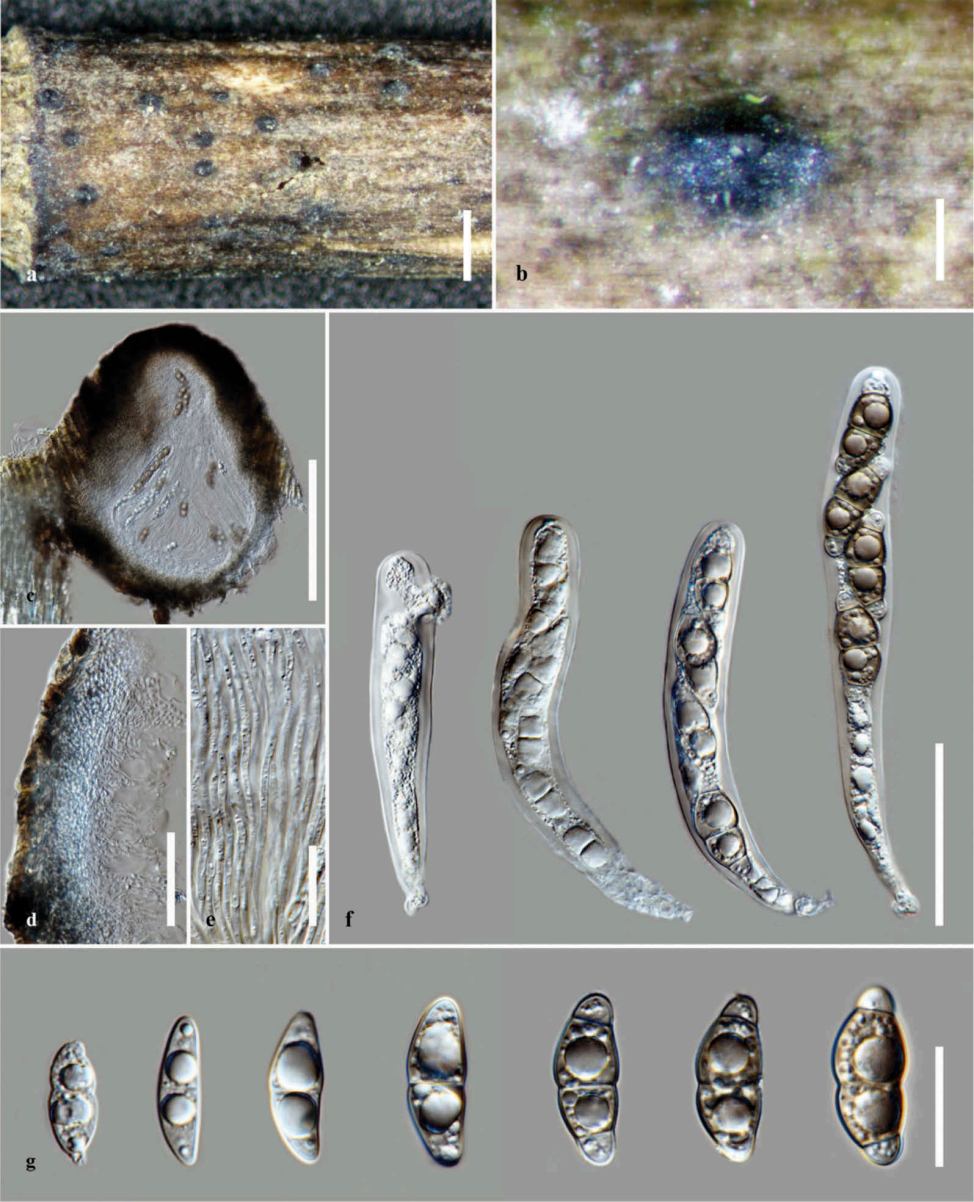
*Halobyssothecium obiones*. (a,b) Appearance of ascomata on *Spartina* culms. (c) Section of ascoma. (d) Section through peridium. (e) Pseudoparaphyses. (f) Asci. (g) Ascospores. Scale bars: a = 1000 μm; b-c = 200 μm; d = 50 μm; e = 20 μm; f = 50 μm; g = 20 μm.

#### Description

*Saprobic* on *Spartina* sp. and other salt marsh halophytes in marine habitats. **Sexual morph**: *Ascomata* 310–410 μm high, 350–420 μm diam. ($\bar{x}$= 360 × 385 μm, n = 5), subglobose or ellipsoidal, immersed to semi-immersed, scattered, ostiolate, carbonaceous, dark brown to black, gregarious. *Papilla* conical, 25–35 μm high, 130–145 μm wide at the apex, composed of several layers of pseudoparenchymatous cells. *Peridium* 30–45 μm wide, comprising two layers: outer layer of brown pseudoparenchyma; inner layer of elongated, hyaline cells. *Pseudoparaphyses* 1.5–2 μm wide, septate, branched. *Asci* 105–155 × 15–20 μm (x̄ = 127 × 17 μm, n = 20), 8-spored, clavate to subcylindrical, short pedicellate with an ocular chamber. Ascospores 20–31 × 6–13 μm (x̄ = 26 × 9 μm n = 20), versicoloured, end cells hyaline, central cells brown, 2-septate at an early stage, 3-septate when mature, and constricted at the septa, slightly curved. **Asexual morph**: Not observed.

#### Material examined

UK, England, Ketch Nature Reserve, Hayling Island, on *Spartina* culms, 24 June 2019, EBG Jones. GJ641.

#### Notes

*Halobyssothecium obiones* is frequently reported on *Spartina* stems, which plays a major role in the breakdown of lignocellulosic secondary walls of plant cells and nutrient recycling (Gessner and Goos 1973; Newell et al. 1995; Barata 2002; Calado et al. 2015, 2019). Our collection of *Halobyssothecium obiones* on *Spartina* sp. culms shares similar morphological characters and overlapping measurements with the recently epitypified *H. obiones* (Dayarathne et al. 2018). However, *Halobyssothecium obiones* observed in this study has shorter ascospore dimensions in contrast to the previous collections (Jones 1962; Cavaliere 1968; Webber 1970), which suggests that *Halobyssothecium* is a complex with at least three or more taxa. Hence, further collections and molecular studies may reveal the complexity in *Halobyssothecium* species.

***Keissleriella phragmiticola*** Wanas., E.B.G. Jones and K.D. Hyde, Fungal Diversity, [43] (2018) Figure 5

**Figure 5.**
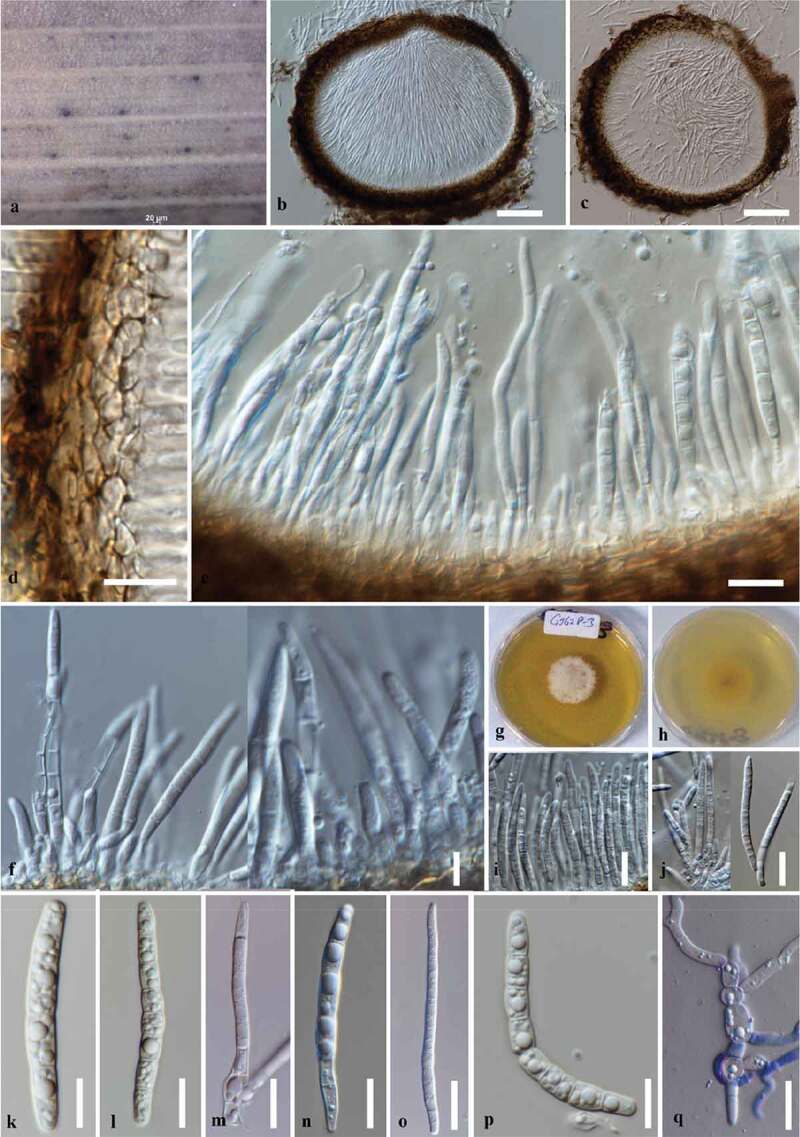
*Keissleriella phragmiticola* (MFLU 19–1194, holotype). (a) Conidiomata on host. (b, c) Sections through the conidiomata. (c, f, i) Conidiogenous cells bearing conidia. (i–p) Multiseptate conidia. q Germinating conidia. g, h Cultures on SMEA (upper, lower). Bars: b, c = 100 µm, d–q = 10 µm.

#### Description

*Saprobic* on *Phragmites communis* in marine habitat appearing as brown to black dots on the host surface. **Sexual morph**: *Keissleriella phragmiticola*. **Asexual morph**: Coelomycetous. *Conidiomata* pycnidial, 105–300 μm high, 1250–320 μm diam. ($\bar{x}$= 195 × 250 μm, n = 10), immersed, dark brown to black, globose to subglobose, slightly depressed, unilocular, ostiolate. *Ostiole* central, short papilla. *Conidiomatal walls* 20–35 μm diam. ($\bar{x}$= 26, n = 10), composed of 5–6 layers of thick-walled, brown to dark brown pseudoparenchymatous cells of *textura angularis*, inner layer with hyaline thick-walled polygonal cells of *textura angularis. Conidiophores* reduced to conidiogenous cells. *Conidiogenous cells* 3–6 × 2.5–4 μm ($\bar{x}$= 4.9 × 2.9 μm, n = 20), hyaline, subcylindrical to dolliform, discrete, determinate, aseptate, smooth, arising from the inner layers of conidioma. *Conidia* 15–50 × 2.5–3.5 μm ($\bar{x}$= 35.9 × 2.9 μm, n = 30), hyaline, cylindrical to subcylindrical, rounded at apex, slightly truncate at base, 5–7-transverse septate, straight to somewhat curved, slightly constricted at the septum, smooth-walled, with one large central guttule in each cell, without sheath and appendages.

#### Culture characteristics

Colonies on MEA, fast growing, reaching 20–35 mm diam., after 10 days of incubation at room temperature, colonies dense, cottony, circular with erose margins, surface raised, colony from above hyaline to cream, below pale yellow without pigmentation.

#### Material examined

UK, Wales, Cardiff Bay Nature Reserve, on woody *Phragmites communis* stem, 15 April 2019. E.B.G Jones. GJ619 (MFLU 19–1194), ex-type living culture MFLUCC 19–0410.

#### Notes

The genus *Keissleriella* comprises 36 species epithets in Index Fungorum 2019. Wanasinghe et al. (2018) described *Keissleriella phragmiticola* on *Phragmites communis* from Poole, Dorset, UK. The species is characterised by superficial to semi-immersed erumpent, globose ascomata and ascospores that are narrowly fusiform with 2–3 large guttules in each cell and surrounded by a thick mucilaginous sheath (Wanasinghe et al. 2018). Our preliminary blast search analyses based on LSU and ITS sequence data of our taxon indicated 99.8% similarity to *Keissleriella phragmiticola* (sexual morph). Hence, we consider our taxon as an asexual morph to *Keissleriella phragmiticola* (sexual morph) and an asexual morph connection is established in this study. *Keissleriella phragmiticola* (asexual morph) resembles *Setoseptoria phragmitis* in having subcylindrical hyaline straight to slightly curved ascospores. However, *Keissleriella phragmiticola* (asexual morph) is distinct from *Setoseptoria phragmitis* in having larger pycnidia and 5–7-transverse septate ascospores. While *Setoseptoria phragmitis* is characterised by smaller conidiomata and 1–3-transverse septate ascospores that are shorter in contrast to *Keissleriella phragmiticola* (Quaedvlieg et al. 2013). The asexual morphs in *Keissleriella* are distinguishable from asexual morph of *Keissleriella phragmiticola* in having cylindrical to bone-shaped hyaline conidia with 0–3-transversesepta (Tanaka et al. 2015).

## Discussion

### Halobyssothecium species complex

Cryptic species are an additional source of undiscovered fungi concealed within previously described taxa or species that are distinct but cannot be readily distinguished based on their morphology (Jones 2011). Among marine fungi, a few genera, such as *Aniptodera, Corollospora, Ceriosporopsis, Halosarpheia, Haiyanga, Saagaromyces* and *Lulworthia* may have cryptic species (Jones 2011; Jones et al. 2015, 2019). Studies on *Halobyssothecium* species have highlighted the confused status of different collections based on morphology which led to the species being placed in various genera and higher taxonomical schemes. Early investigations indicated two morphologically similar taxa, one with ascospore measurements in the range 24–38 × 8–14 μm, while others measured 38–56 × 16–22 μm (Jones 1962; Cavaliere 1968; Webber 1970). Our new collection introduces a third sexual morph with similar ascospore measurements to *Halobyssothecium obiones* but which phylogenetically differs by 5.1% nucleotide base pair differences in ITS and 2.9% in TEF gene regions. Unfortunately, no cultures or molecular data are available for *Halobyssothecium* collections with the larger ascospore measurements. Cribb and Cribb (1960) reported *Halobyssothecium* obiones-like species with smaller ascospores from mangrove habitats in Australia, which may be a misidentification as most collections of this species complex are known from temperate salt marsh locations (Kohlmeyer and Kohlmeyer 1979).

DNA sequence data has revealed several cryptic taxa and allowed us to understand evolutionary relations in the fungi as phenotypic characters alone may not be adequate for species identification (De Gruyter et al. 2009, 2013). Jones et al. (2015, 2017)) introduced a number of new marine genera for *Aniptodera* species: *Praelongicaulis kandeliae* (= *A. kandeliae*), *Paraaniptodera longispora* (= *A. longispora*) and *Aniptosporopsis lignatilis* (= *A. lignatilis*) based on phylogenetic studies. The marine genus *Lulworthia* also poses a major taxonomical challenge in that all species have filiform hyaline ascospores with a polar chamber from which a drop of mucilage is released (Cavaliere and Johnson 1966; Jones 1994; Kohlmeyer et al. 2000). Segregation has only been resolved by molecular studies or the possession of an asexual morph: *Lulwoana uniseptata* (= *Zalerion maritimum*) (Abdel-Wahab and Bahkali 2012).

In case of cryptic species or complex genera with morphologically similar species, asexual stages play a vital role in demarcating species (Shenoy et al. 2007). Earlier reports showed that most *Halobyssothecium* species produced a phoma-like asexual morph. However, several studies on halobyssothecium-like species have not accurately described their asexual morphs (Calado and Barata 2012; Dayarathne et al. 2018). We have found a xylomyces-like asexual morph which is connected to the *Halobyssothecium estuariae* sexual morph which supported in establishing a distinction from *H. obiones*. We presume that precise morphological characterisation of both sexual and asexual morphs might assist in delimiting *Halobyssothecium* species. Some 50% of all marine fungi described have no sequence data (Hassett et al. 2019), therefore further collection and isolation are required, and this will assist in identifying other cryptic species and avoid misidentification.
